# Primate-to-Human Retroviral Transmission in Asia

**DOI:** 10.3201/eid1107.040957

**Published:** 2005-07

**Authors:** Lisa Jones-Engel, Gregory A. Engel, Michael A. Schillaci, Aida Rompis, Artha Putra, Komang Gde Suaryana, Agustin Fuentes, Brigitte Beer, Sarah Hicks, Robert White, Brenda Wilson, Jonathan S. Allan

**Affiliations:** * University of Washington National Primate Research Center, Seattle, Washington, USA;; † Swedish/Providence Hospital Family Practice Residency, Seattle, Washington, USA;; ‡ University of Toronto at Scarborough, Toronto, Ontario, Canada;; § Udayana University, Denpasar, Bali, Indonesia;; ¶ University of Notre Dame, Terre Haute, Indiana, USA;; # Southern Research Institute, Frederick, Maryland, USA;; ** Southwest Foundation for Biomedical Research, San Antonio, Texas, USA

**Keywords:** Disease Transmission, Simian foamy virus, exogenous retrovirus, primate zoonoses, Macaca, Southeast Asia

## Abstract

We describe the first reported transmission to a human of simian foamy virus (SFV) from a free-ranging population of nonhuman primates in Asia. The transmission of an exogenous retrovirus, SFV, from macaques (*Macaca fascicularis*) to a human at a monkey temple in Bali, Indonesia, was investigated with molecular and serologic techniques. Antibodies to SFV were detected by Western blotting of serum from 1 of 82 humans tested. SFV DNA was detected by nested polymerase chain reaction (PCR) from the blood of the same person. Cloning and sequencing of PCR products confirmed the virus's close phylogenetic relationship to SFV isolated from macaques at the same temple. This study raises concerns that persons who work at or live around monkey temples are at risk for infection with SFV.

Recent epidemics such as HIV and severe acute respiratory syndrome (SARS) have changed the way we view emerging infectious diseases; these epidemics show that animal reservoirs are important sources of new infectious threats to humans. Contact between humans and animals is a crucial rate-limiting step in this process, although data describing the variables that influence animal-to-human transmission are relatively scarce. Nonhuman primates, by virtue of their genetic, physiologic, and sometimes social similarities to humans, are particularly likely sources of infectious agents that pose a threat to humans (1,2). Data on simian immunodeficiency virus (SIV)/HIV dramatize this point; scientists now theorize that SIVs were transmitted from primates to humans on several occasions (3,4). As a result, concern is increasing that other infectious agents enzootic in primate populations may endanger humans (5).

The family of SIV is 1 of 4 primateborne retroviruses known to infect humans (6). Simian T-cell lymphotropic viruses, enzootic in both Asian and African Old World monkeys and apes, may have repeatedly crossed the species barrier (7,8). The resulting human form of the virus, HTLV, is the etiologic agent of 2 human diseases, adult T-cell leukemia and tropical spastic paresis (9). Serologic studies have demonstrated evidence of primate-to-human transmission of simian retrovirus (SRV), a retrovirus enzootic among Old World monkeys, in laboratory workers exposed to captive primates (10). To date, no disease has been linked to human infection with SRV. Finally, in the past decade, evidence of infection with simian foamy virus (SFV) has been identified in 1% to 4% of persons who come into frequent contact with primates in zoos and primate laboratories and among 1% of bushmeat hunters in Central Africa (11–15).

## SFV

SFVs are exogenous retroviruses enzootic in both New and Old World primates (16–18). Phylogenetic analyses of SFVs indicate a species-specific distribution of virus strains not unlike that of SIV among some African primate species (19–23). Among captive primate populations, seroprevalence of antibodies to SFV may reach 100% in adults, with many animals seroconverting before the onset of sexual maturity (19,24; J. Allan, unpub. data). Fewer data are available on the seroprevalence of antibodies to SFV among free-ranging populations of primates. SFV is present in highest concentrations in the saliva of infected laboratory macaques (*Macaca mulatta* and *M*. *fascicularis*) and African green monkeys (*Cercopithecus aethiops*), which suggests that bites, scratches, and mucosal splashes with saliva from primates are likely mechanisms of transmission (25–27).

Because SFV is not known to occur naturally in humans, detecting serologic or molecular evidence, or both, of infection in a human, along with a history of close contact with primates, constitutes strong evidence for primate-to-human transmission, i.e., a marker for cross-species transmission (28). Existing serologic and molecular techniques can sensitively and specifically detect human SFV infections (29).

Primates and humans come into contact in a variety of contexts in Asia, including owning primate pets, observing performance monkeys, participating in ecotourism activities, hunting primates for bushmeat, and visiting monkey temples (30,31). Monkey temples are religious sites that have, over time, become associated with populations of free-ranging primates. Monkey temples are common throughout southern and Southeast Asia, where primates play an important role in culture. Asia's monkey temples annually bring millions of people, including hundreds of thousands of tourists, from around the world into close proximity with free-ranging primates (32–34). Worldwide, monkey temples may account for more human-primate contact than any other context.

In Bali, ≈45 temple sites contain substantial populations of free-ranging macaques (33). Those who spend the most time in or around monkey temples include workers who maintain the temples; nuns, monks, and others who live on or around temple grounds; merchants who sell a variety of goods to tourists; and farmers whose fields are raided by macaques. Worshippers and tourists may also come into contact with temple macaques. These temples are thus an important context in which to investigate cross-species transmission of infectious agents.

Serologic data on free-ranging Southeast Asian macaques, though incomplete, suggest that SFV is enzootic among these macaque populations and that corresponding rates of SFV infection are high (80%–100%) (L. Jones-Engel, unpub. data). We hypothesized that humans who come into contact with these macaques might similarly show evidence of infection with SFV and investigated this proposition among a group of persons who worked at or around the Sangeh monkey temple in Bali, Indonesia.

## Methods

### Study Site: Sangeh Monkey Temple

The Sangeh monkey temple is in central Bali, Indonesia ≈20 km north of Denpasar, Bali's most populous city. The 17th-century Hindu temple complex at Sangeh serves the surrounding community and is a popular domestic and international tourist destination. Approximately 200 free-ranging *M*. *fascicularis* roam throughout the 6-hectare temple complex and into the surrounding rice fields and farms. Most of the macaques' caloric intake is from daily provisions provided by temple workers and food given to them by visitors.

### Human Participant Sampling

In July 2000, as part of a larger study on the epidemiology of exposure to primateborne viruses at the Sangeh monkey temple, 82 workers from Sangeh agreed to participate in the present study (32). After informed consent was obtained, a questionnaire designed to elicit demographic data as well as data describing the frequency and type of exposure to Sangeh's macaques was administered in Bahasa Indonesia, the national language of Indonesia. Subsequently, 10 mL of blood was withdrawn from each participant's antecubital vein, 6 mL was centrifuged to extract serum, and the remainder was mixed with EDTA. Serum specimens and whole blood were then stored at –20°C.

### Macaque Sampling

In July 2000, 38 macaques within the Sangeh monkey temple area and surrounding forest were darted opportunistically and sedated with 3 mg/kg of Telazol (tiletamine HCl/zolazepam HCl). Following universal precautions, researchers withdrew 10 mL of blood from each macaque's femoral vein. The macaques were closely monitored during anesthesia and recovery. Six milliliters of blood was placed in a serum separator tube and centrifuged in the field to extract the serum. The remaining blood was placed in a tube containing EDTA. Sera and whole blood were frozen and stored at –20°C.

### Western Blot Analysis

Western blot immunoassays were performed with a few modifications (21). Briefly, human foreskin fibroblast cells were infected with SFVbab1 (an isolate from a baboon) and maintained until notable cytopathologic changes were observed (19). Culture supernatant fluid containing virus was harvested, and SFV was purified through a 20% sucrose cushion, separated by sodium dodecyl sulfate–polyacrylamide gel electrophoresis, and the antigens were blotted onto nitrocellulose sheets. The nitrocellulose paper was blocked with 3% bovine serum albumin and subsequently incubated with serum at a dilution of 1:40. Viral proteins were detected with the streptavidin-biotin system (Amersham Inc., Arlington Heights, IL, USA) by using diaminobenzidine as the substrate for color development. The criterion used for a positive sample was antibody reactivity to both p70 and p74 *gag*-related proteins (19).

### PCR Detection of SFV

DNA was purified from whole blood by using the QIAamp DNA Blood Mini Kit (QIAGEN, Inc., Valencia, CA, USA). Briefly, 20 μL protease and 200 μL buffer AL were combined with 200 μL whole blood and incubated at 56°C for 10 min. After incubation, 200 μL ethanol was added, and the entire mixture was applied to a QIAamp spin column. The purified DNA was eluted from the column with 70 μL nuclease-free water, and concentration was determined spectrophotometrically at optical density 260 nm.

The presence of SFV DNA was determined by using nested PCR. Five hundred ng purified DNA was combined with a PCR reaction mixture with a final concentration of 10 mmol/L Tris (pH 9.0), 50 mmol/L KCl, 0.1% Triton X-100, 2 mmol/L MgCl_2_, 200 μmol/L each dNTP, 0.15 mg/mL BSA, 1 U Taq polymerase, and 400 nmol/L of each primer in a total volume of 50 μL. The following primer pairs were used: first round, forward 5´ CAG TGA ATT CCA GAA TCT CTT C 3´, reverse 5´ CAC TTA TCC CAC TAG ATG GTT C 3´; and second round, forward 5´ CCA GAA TCT CTT CAT ACT AAC TA 3´, reverse 5´ GAT GGT TCC CTA AGC AAG GC 3´ (29). "Touchdown PCR" was used for both rounds with reaction conditions of initial denaturation at 94°C for 2 min, followed by 7 cycles of 94°C for 30 s, 60°C for 30 s, and 72°C for 45 s, with a 2°C decrease in annealing temperature per cycle to 48°C, followed by 33 cycles of 94°C for 30 s, 48°C for 30 s, and 72°C for 45 seconds with a final extension at 72°C for 2 min. Second-round conditions were the same, except 19 cycles were used instead of 33.

Twenty μL of the PCR reaction underwent electrophoresis on a 1% low melting point agarose gel. DNA bands (365-bp product) were excised from the gel and purified by using Wizard PCR Preps DNA Purification System (Promega Corp., Madison, WI, USA). The PCR product was ligated into the pCR 2.1-TOPO vector by using the TOPO TA Cloning Kit (Invitrogen Corp., Carlsbad, CA, USA.). An SFV DNA plasmid (pSFV-1Lgp), representing long terminal repeat, *gag*, and *pol* of SFV-1, was included as a positive PCR control and for determining sensitivity of detection by serial dilution (provided by A. Mergia). TOP10 cells were transformed with the ligation reaction, plated onto Luria broth agar plates containing 50 μg/mL kanamycin, and incubated overnight at 37°C. Miniscreen DNA was purified by using Wizard Plus Minipreps DNA Purification System (Promega). Samples were sequenced with the ABI 373 automated fluorescent sequencer using BigDye Terminator cycle sequencing chemistry (Applied Biosystems, Foster City, CA, USA).

### Amplification of Mitochondrial Sequences

Five hundred ng purified DNA from whole blood was combined in a PCR reaction mixture with a final concentration of 10 mmol/L Tris (pH 9.0), 50 mmol/L KCl, 0.1% Triton X-100, 2.5 mmol/L MgCl_2_, 200 μmol/L each dNTP, 0.15 mg/mL BSA, 1 μm Taq polymerase, and 400 nmol/L of each primer in a total volume of 50 μL. The following primers were used: forward, 12SA, 5´ CTG GGA TTA GAT ACC CAC TAT 3´, and reverse, 12SO, 5´ GTC GAT TAT AGG ACA GGT TCC TCT A 3´ (35). Cycling conditions were the following: initial denaturation at 94°C for 5 min, followed by 35 cycles of 94°C for 30 s, 55°C for 30 s, and 72°C for 5 min, with a final extension at 72°C for 5 min. The 101-bp product underwent electrophoresis and was processed for DNA sequencing essentially as described for SFV.

The alignments were made in Bioedit (http://www.mbio.ncsu.edu/BioEdit/bioedit.html) and ClustalX 1.81 (ftp://ftp-igbmc.u-strasbg.fr/pub/ClustalX/). Columns in the alignment in which gaps had been inserted in regions with insertions, and deletions were stripped before the analyses. DNA trees were created with the neighbor-joining method by using the Phylip program (DNAdist; Neighbor), and the output was generated with Treeview (http://taxonomy.zoology.gla.ac.uk/rod/treeview.html). The GenBank accession numbers for the SFV and mitochondrial DNA sequences reported here are AY628152-69 and AY633510-39, respectively.

## Results

### Seroprevalence of SFV among Macaques

The seroprevalence of SFV among the Sangeh macaques is presented in Table 1. Thirty-eight macaques (29 males and 9 females; 4 juveniles, 6 subadults, 28 adults) were sampled. Thirty-four (89.5%) of the 38 macaques were seropositive for SFV by Western blot; 2 (50%) of 4 juveniles, all 6 (100%) subadults, and 26 (93%) of the adults were antibody positive. All 9 females were SFV seropositive. SFV seroprevalence in this free-ranging macaque population was consistent with seroprevalence studies done in captive (25) and other free-ranging macaque populations (L. Jones-Engel, unpub. data).

**Table 1:** Seroprevalence of antibodies to simian foamy virus among Sangeh macaques (Macaca fascicularis)

Age group*	No. Western blot positive/total (%)
Juvenile	2/4 (50.0)
Male	2/4 (50.0)
Subadult	6/6 (100.0)
Male	4/4 (100.0)
Female	2/2 (100.0)
Adult	26/28 (92.9)
Male	19/21 (90.5)
Female	7/7 (100.0)
All ages	34/38 (89.5)
Male	25/29 (86.2)
Female	9/9 (100.0)

*Juveniles are defined as 1–3 years of age, subadults as 3–5 years of age, adults as >5 years of age.

### Human Participant Exposure Data

Nearly two thirds of the study sample was male (62.2%), and the mean age of the study participants was 35 years (Table 2). Of participants who reported being exposed, 23 (28.0%) of 82 persons reported having been bitten by the temple's macaques (Table 3). Five of the 23 reported being bitten more than once. Thirty-one (37.8%) of 82 reported having been scratched, including 9 persons who had been scratched more than once. In total, 37 (45.1%) of 82 reported having been either bitten or scratched.

**Table 2:** Human study population demographic characteristics

Demographic characteristic	No. (%)
All participants	82 (100.0)
Age group (y)	
<20	9 (11.0)
20–29	23 (28.0)
30–39	20 (24.4)
40–49	22 (26.8)
>49	8 (9.7)
Sex	
Male	51 (62.2)
Female	31 (37.8)

**Table 3:** Prevalence of reported bite and scratch injuries

Descriptor	No. (%)
Bitten	23 (28.0)
Bitten more than once	5 (6.1)
Scratched	31 (37.8)
Scratched more than once	9 (11.0)
Bitten or scratched	37 (45.1)
Possessed food at time of injury	35 (94.6)
All participants	82 (100.0)

**Figure 1:**
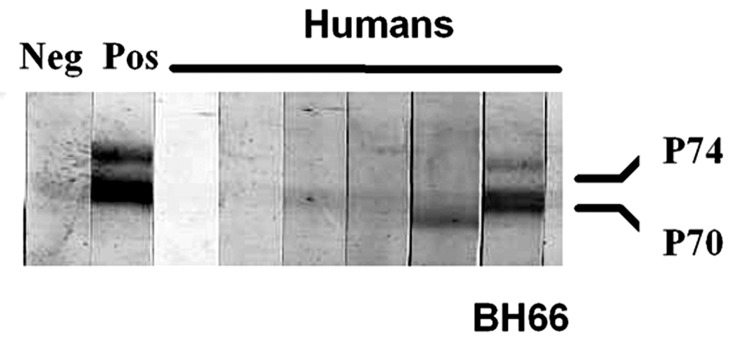
Western blot analysis of human serum samples for evidence of simian foamy virus (SFV) antibodies. Antibodies to the *gag* precursor proteins (p70/p74) were apparent from the human BH66 blood sample, which indicated infection with SFV. Positive control is an SFV-infected baboon.

Of the 82 persons whose serum was tested for antibodies to SFV, 81 had negative results by Western blot. Serum from a 47-year-old farmer (BH66) was reactive to SFV gag proteins, p70/p74 (Figure 1). This man reported that he visited the monkey temple every day and had previously been bitten once and scratched on more than one occasion by macaques there. He reported that the bite and scratches, which occurred on his hands and toes, had bled and that he had washed the wounds and applied traditional medicines. He denied owning or coming into contact with a pet primate. He also denied hunting primates or consuming primate meat.

### Detection of SFV DNA in a Person from Bali

To determine whether SFV was present in humans and macaques, we performed nested PCR amplification of SFV by using conserved primers designed to detect macaque SFV (29). SFV was detected in the macaques tested, as well as in the 1 human participant (BH66), whose serum contained antibodies to SFV. Blood samples from all 81 human participants that were seronegative for antibodies to SFV were also negative for SFV by PCR. The limits of sensitivity for nested PCR were 1–10 SFV DNA copies per 500 ng cellular DNA, as determined by dilution of a positive control plasmid.

**Figure 2:**
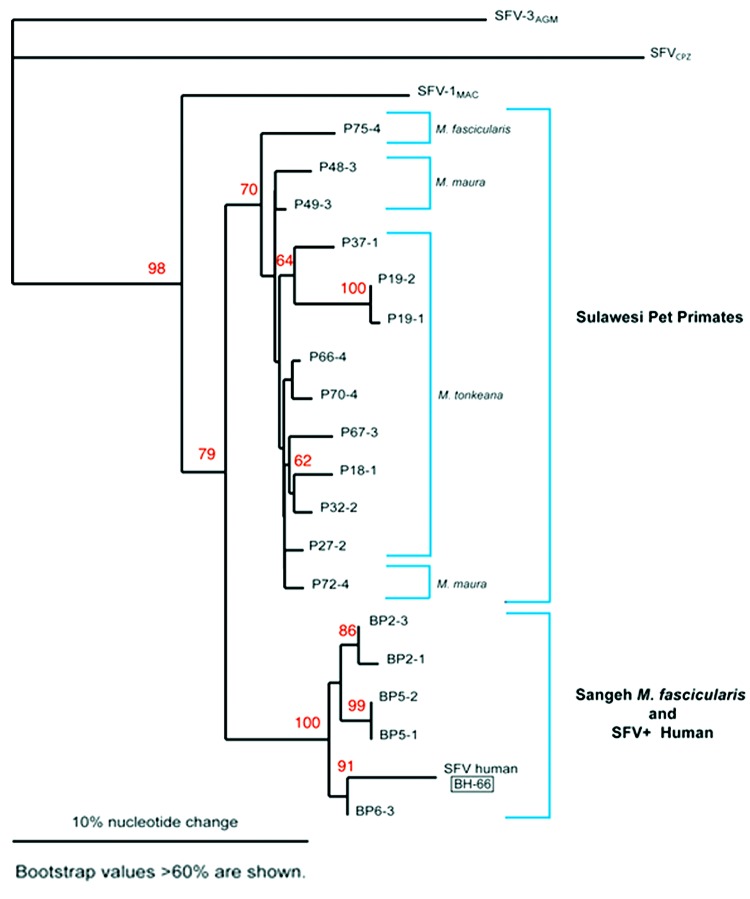
Phylogenetic analysis of simian foamy virus (SFV) DNA from several species of Indonesian primates and an infected human. BP 2, 5, and 6 represent Sangeh monkey temple macaques (*Macaca fascicularis*). P 18, 19, 27, 32, 37, 66, 67, and 70 are pet macaques (*M*. *tonkeana*) from Sulawesi, Indonesia. P48, 49 and 72 are pet macaques (*M*. *maura*) from Sulawesi, Indonesia. P75 is a pet *M*. *fascicularis* macaque from Sulawesi, Indonesia. All Sulawesi pet primate samples were collected during 2000. SFV-1mac represents a published sequence from a rhesus macaque (*M*. *mulatta*), and SFV-3agm is a published sequence from an African green monkey (*Cercopithecus aethiops*). SFVcpz is a published sequence from a chimpanzee (*Pan troglodytes*) and was used an outgroup for this tree. The SFV human strain (BH66) clustered with an SFV sequence amplified from BP6 one of the macaques at the Sangeh monkey temple. The SFV DNA tree was created with the neighbor-joining method by using the PHYLIP program (DNAdist; Neighbor). Bootstrap replicates were 1,000. Bootstrap values were calculated by using Seqboot, DNAdist, Neighbor, and Consense (PHYLIP programs). Bootstrap values >60% are shown. The SFV tree was plotted in Treeview.

**Figure 3:**
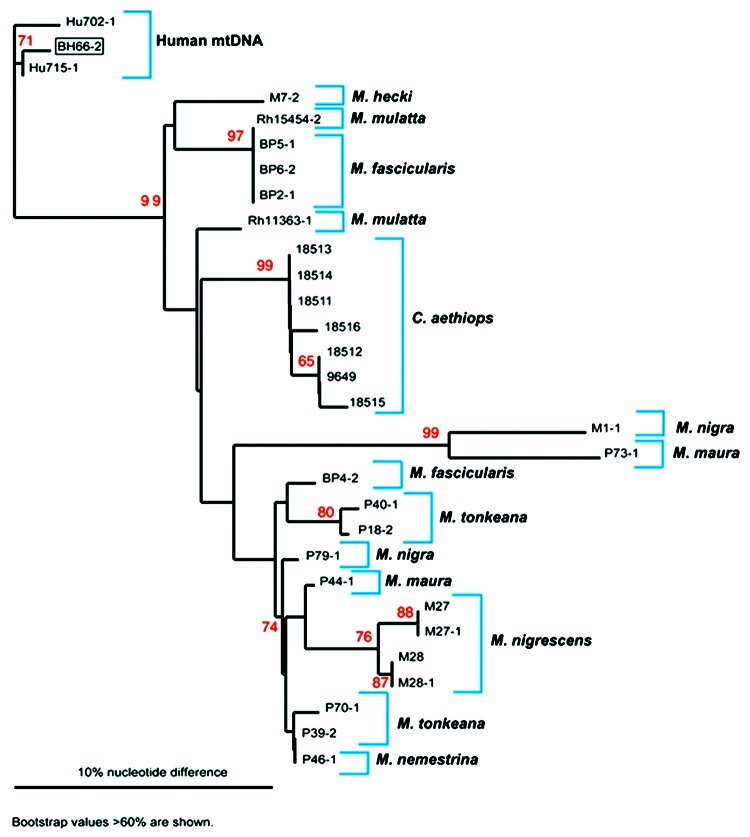
Phylogenetic analysis of mitochrondrial (mt) DNA from nonhuman primates and humans. mtDNA was amplified and sequenced from the simian foamy virus–infected person (BH66), 2 human controls (Hu702 and Hu715), *M*. *mulatta* (Rh15454, 18511,18512, 18513,18514,18515, 11363, 9649), *M*. *fascicularis* (BP2, 4, 5, 6), *M*. *nemestrina* (P46), *M*. *tonkeana* (P18,39,40), *M*. *maura* (P44, 73), *M*. *nigra* (P79, M1); *M*. *nigrescens* (M27, 28), and *M*. *hecki* (M7). The mtDNA tree was created with the neighbor-joining method with the Phylip program (DNAdist; Neighbor). Bootstrap replicates were 1,000. Bootstrap values were calculated by using Seqboot, DNAdist, Neighbor, and Consense (PHYLIP programs). Bootstrap values >60% are shown. The mtDNA tree was plotted in Treeview. This analysis suggests that BH66 was of human origin. Although the phylogenetic tree constructed with mtDNA from a variety of monkey samples can be used to distinguish human from monkey mtDNA, a large number of nuclear mtDNA sequences, have evolved as pseudogenes (36). These sequences can be highly divergent from mtDNA and resulted in some ambiguity as mtDNA amplified from several monkeys did not group with other members of the same species. Because of the nature and variability of these sequences, definitive conclusions about mtDNA phylogenies could not be determined; however, mtDNA trees were still useful for determining the origin of mtDNA material.

Quantitative PCR products of BH66, 5 of the Sangeh macaques (*M*. *fascicularis*), and 13 pet macaques (*M*. *tonkeana*, *M*. *maura*, and *M*. *fascicularis*) from Sulawesi, Indonesia, were cloned at least twice, sequenced, and compared with published sequences from a rhesus macaque (*M*. *mulatta*) (SFV-1mac, M55279), an African green monkey (*Cercopithecus aethiops*) (SFV-3agm, M74895), and a chimpanzee (*Pan troglodytes*) (SFVcpz, U04327). As shown in Figure 2, SFV from BH66 was most closely related to an SFV sequence amplified from 1 of the macaques at the Sangeh Monkey Temple (BP6). To verify that the BH66 sample was of human origin and not a mislabeled sample from an SFV-infected monkey, we amplified a small fragment from the 12S ribosomal mitochondrial DNA from 2 healthy unexposed humans, BH66, several macaque species, and African green monkeys; cloned the products; and derived DNA sequences. Phylogenetic analysis (Figure 3) showed that the human DNA sample grouped with mitochondrial DNA sequences from humans, confirming that the BH66 sample was from a human. Lymphocytes from BH66 were not available for isolation of SFV directly.

## Discussion

### Human Infection with SFV at the Sangeh Monkey Forest

This report documents the first case of SFV infection in a person with known exposure to free-ranging Asian primates. Antibodies to SFV were detected in serum, and SFV genomic segments were amplified from blood of the infected person. Because PCR is prone to contamination, which leads to false-positive results, PCR products were sequenced and compared to SFV from other macaque species. Phylogenetic analysis showed that SFV amplified from the infected human was most similar to SFV from *M*. *fascicularis* at the Sangeh monkey temple. Although ascertaining exactly how or when the person acquired his infection was not possible, he did report having been bitten once and scratched on a number of occasions by macaques at Sangeh. He denied other past contacts with primates.

Because only 1 infected person was detected and our sample size was limited (82 persons), we cannot estimate the prevalence of SFV infection in this human sample. Previous research on naturally acquired SFV infection among bushmeat hunters in Central Africa found 10 who seroconverted in a population of 1,099 (1%), of which 3 also had positive results by PCR. Surveillance of larger numbers of persons exposed to primates at monkey temples is necessary to estimate the risk of SFV infection in this context.

### Monkey Temples in Bali as Contexts for Primate-Human Contact

Human and macaque sympatry in Southeast Asia dates back as far as 25,000 years, as evidenced by remains at Niah Cave in Borneo (37). Hindu and Buddhist temples are a relatively recent addition to the landscape, first appearing 1,000 to 4,000 years ago. Contemporary human-macaque commensalism at each monkey temple is shaped by the behavioral characteristics of the particular monkey population as well as the community's unique geographic, social, religious, and economic factors. Human-macaque contact differs at the various monkey temples. At Sangeh, where tourism has become an important economic resource, macaques depend on visitor feeding for most of their nutrition and have learned to climb on visitors' heads and shoulders to obtain food. Local photographers, who make a living photographing visitors with monkeys, encourage this behavior. Macaques, sometimes provoked by visitors, can become aggressive and will bite or scratch people.

The Sangeh Temple Committee, which manages the Sangeh Monkey Forest, estimates ≈250 persons work in and around the monkey forest. Of the 82 persons who participated in the present study, 28% had been bitten by a macaque, and 6% had been bitten more than once. In comparison, 700,000 tourists visit the 4 main monkey temples on the island of Bali (Padangtegal/Ubud, Sangeh, Alas Kedaton, and Uluwatu) annually and as many as 5% of these visitors are bitten by macaques (A. Fuentes, unpub. data). These data suggest that visitors receive the preponderance of macaque bites.

### Importance of SFV Infection in Humans

The literature describes ≈40 cases of human infection with SFV. Long-term follow-up data are unavailable for many of these cases. However, no disease has been linked to SFV among infected humans. Furthermore, no cases of human-to-human transmission have been described, even among those receiving transfusions of blood products from a worker at a primate center who was later shown to be infected with SFV (38).

Notwithstanding, more data are needed before SFV can be proclaimed a "virus without a disease." First, SFV infection has not been extensively studied in immunocompromised persons, so whether SFV has a more aggressive course in an immunologically "permissive" environment is unknown. Two persons died shortly after receiving baboon liver transplants in 1994, and SFV was detected in the blood and tissues of both at autopsy (39). More research in this area is needed. Moreover, little is known about the epizootiology of SFV among wild primates. Although SFV infection in humans has not culminated in any observable symptoms, SFV strains may differ in their capacity to infect or cause disease in humans. Finally, whether SFV can adapt in humans after transmission and potentially lead to disease needs to be examined.

### Implications and Recommendations for Public Health

The recent SARS epidemic vividly demonstrates how the economic infrastructure and dense population of Asia facilitate the rapid international spread of disease. The combination of large primate reservoirs, prevalent human-primate contact, a growing immunocompromised population, and advanced infrastructure in Asia increases the likelihood of a primateborne zoonosis emerging on this continent.

The demonstration of SFV transmission in the context of a monkey temple in Bali points to a broad public health concern: other enzootic primate infectious agents may cross the species barrier and cause significant morbidity and mortality in human populations. Given our lack of knowledge of the effects of SFV, as well as the poorly defined risk of other primateborne zoonoses, steps should be taken to decrease the risks of cross-species transmission among the many persons who visit these sites. Previous data on both worker and visitor exposure to macaques at monkey temples suggest that human behaviors, especially the practice of feeding macaques, is a risk factor for being bitten or scratched (33,34). We have recommended that monkeys be fed only by specially trained personnel who minimize physical contact with monkeys. Such restrictions have been successfully employed at other monkey forests in Asia. For example, Singapore's Bukit Timah Nature Reserve has nearly 200 free-ranging macaques (*M*. *fascicularis*), and in 2002, an estimated 380,000 visitors made use of the reserve's trail system, yet contact between monkeys and visitors at Bukit Timah is rare. This finding may be ascribed to the park's policy, enforced by stiff fines, of prohibiting visitors from feeding macaques.

## Conclusions

This study reports the first case of human SFV infection in Asia and also, for the first time, links natural transmission of SFV to a person to a specific population of primates. Our findings suggest that workers in and around monkey temples can become infected with SFV. By implication, visitors to monkey temples, especially those who are bitten by a macaque, may also be at risk for SFV infection. Because many visitors to monkey temples are international travelers, these findings have ramifications for the potential global spread of primateborne infectious agents. Demonstrating natural cross-species transmission in a context that does not involve hunting for bushmeat implies that other contexts of primate-human contact may also facilitate the transmission of simian pathogens. These data point to the need for further research into SFV transmission in other contexts, including pet ownership and performance animals, as well as in the diverse geographic areas where humans and primates come into contact. Such research will help describe the overall picture of the emergence of primateborne pathogens such as HIV/SIV and will form a scientific basis for guiding policies and programs to deter the spread of emerging zoonotic pathogens.
